# A practical predictive model to predict 30-day mortality in neonatal sepsis

**DOI:** 10.1590/1806-9282.20231561

**Published:** 2024-08-16

**Authors:** Tengfei Qiao, Xiangwen Tu

**Affiliations:** 1 Nanjing Lishui District Hospital of Traditional Chinese Medicine, Department of Laboratory Medicine - Nanjing, China.; 2 GanZhou Women and Children's Health Care Hospital, Department of Laboratory Medicine - Ganzhou, China.

**Keywords:** Hemoglobin, Model, Mortality, Neonatal sepsis, Prothrombin time

## Abstract

**OBJECTIVE:**

Neonatal sepsis is a serious disease that needs timely and immediate medical attention. So far, there is no specific prognostic biomarkers or model for dependable predict outcomes in neonatal sepsis. The aim of this study was to establish a predictive model based on readily available laboratory data to assess 30-day mortality in neonatal sepsis.

**METHODS:**

Neonates with sepsis were recruited between January 2019 and December 2022. The admission information was obtained from the medical record retrospectively. Univariate or multivariate analysis was utilized to identify independent risk factors. The receiver operating characteristic curve was drawn to check the performance of the predictive model.

**RESULTS:**

A total of 195 patients were recruited. There was a big difference between the two groups in the levels of hemoglobin and prothrombin time. Multivariate analysis confirmed that hemoglobin>133 g/L (hazard ratio: 0.351, p=0.042) and prothrombin time >16.6 s (hazard ratio: 4.140, p=0.005) were independent risk markers of 30-day mortality. Based on these results, a predictive model with the highest area under the curve (0.756) was built.

**CONCLUSION:**

We established a predictive model that can objectively and accurately predict individualized risk of 30-day mortality. The predictive model should help clinicians to improve individual treatment, make clinical decisions, and guide follow-up management strategies.

## INTRODUCTION

Neonatal sepsis is a serious and life-threatening disease that needs timely and immediate medical attention^1^. Despite advances in medical care, neonatal sepsis remains a ­significant cause of ­morbidity and mortality in neonates worldwide. The ­prevalence of neonatal sepsis and mortality rates varies across different regions and healthcare settings, with higher rates reported in ­low-resource areas^2^. Studies have reported that various ­biomarkers can affect the prognosis of ­neonatal sepsis, ­including gestational age, birth weight, presence of ­comorbidities, blood change, and the type of infecting ­organism^3–7^. Early ­identification and suitable treatment are essential for ­improving outcomes in patients with sepsis. To neonates, the clinical ­manifestations of sepsis can be ­nonspecific. Laboratory investigations, such as blood pressure ­monitoring, interleukin-18, and elevated ­neutrophil-to-monocyte ratio, are helpful for clinicians to ­evaluate the risk of adverse ­outcomes and guide treatment ­decisions^8–10^. So far, there is no specific prognostic biomarkers or model for dependable ­predict outcomes in neonatal sepsis. Our goal is to establish a ­predictive model based on readily available laboratory data to assess 30-day mortality in neonatal sepsis.

### Patients

The investigation recruited neonates who were admitted to the hospital between January 2019 and December 2022. The ­admission information was obtained from the medical record retrospectively. The neonates were limited to those who were ­diagnosed with neonatal sepsis, with complete patient ­information, and aged within 28 days. Finally, 195 patients were recruited in the cohort. The diagnosis of neonatal sepsis was based on the International Consensus on Pediatric Sepsis definition^11^. The study was approved by the ethics committee at the local hospital and was conducted in accordance with the guidelines set out in the Declaration of Helsinki.

### Data

The following admission indexes were gathered in this ­investigation: age, gender, gestational age, weight, Apgar scores (5 min and 10 min), admission laboratory results, ­including routine blood test (neutrophils, lymphocyte, ­monocyte, ­platelet, ­hemoglobin), ­biochemical indicators [alanine ­aminotransferase (ALT), ­aspartate transaminase (AST), lactate dehydrogenase (LDH), direct ­bilirubin (DB), albumin (ALB), urea ­nitrogen (UREA), ­creatinine (CREA)], and coagulation function [­prothrombin time (PT), ­international normalized ratio (INR), activated partial ­thromboplastin time (APTT) and thrombin time (TT)]. The primary outcome of ­neonatal sepsis was 30-day mortality, and the primary outcome was obtained from ­medical records or by telephone.

### Statistical analysis

All statistical analyses were conducted with SPSS 21.0 (SPSS, Inc., IA, USA). Continuous variables, shown as ­mean±standard deviation, were compared with t-test and analysis of ­variance (ANOVA). Categorical variables (numbers and ­percentages) were compared using the chi-square test. Univariate or ­multivariate analysis was utilized to identify independent risk factors for 30-day mortality. Patients were divided into two groups ­according to the primary outcome. The receiver operating characteristic (ROC) curve was drawn to check the performance of the model in predicting the primary events and confirmed the optimal cutoff value of the predictive model. All tests were two-sided, and p-values <0.05 were considered statistically significant.

## RESULTS

### Baseline characteristics

The basic information of patients is presented in Table 1. Of these patients, the 30-day mortality rate was 17.5%. There were 123 males and 72 females, with a median age of 3 days (­ranging from 1 day to 28 days). Compared with the ­nonsurvivor group, the significant elevated levels of platelet and ALB and the ­significant declined levels of ALT, AST, UREA, PT, INR, and TT were found in the survivor group (all p<0.05). Beyond that, obvious difference was also found in the numbers of ­culture positive, the levels of hemoglobin, LDH, DB, and CREA between the two groups, although statistical significance was not reached (all p<0.10) (Table 1).

**Table 1:** Characteristics of patients.

General	Survivor (n=166)	Nonsurvivor (n=29)	p-value
Gender (male/female), n	106/60	s17/12	0.521
Median age (range), days	3 (1, 27)	5 (1, 28)	0.407
Gestational age at birth (weeks)	38.5 (29.0, 41.0)	38.0 (27, 41)	0.671
Weight (g)	3050 (1090, 3860)	750.19 (1088, 3510)	0.351
Apgar score (1 min)	10 (5, 10)	9 (4.5, 10)	0.570
Apgar score (5 min)	10 (8, 10)	10 (9, 10)	0.299
Culture positive, n (%)	50 (30.1)	6 (20.1)	0.070
CRP (mg/L)	7.85 (0.1, 96.4)	20.6 (0.1, 152.3)	0.187
Monocyte (×109/L)	0.92 (0.14, 2.96)	0.77 (0.13, 4.18)	0.655
Lymphocyte (×109/L)	3.66±1.80	4.23±3.62	0.309
Neutrophil (×109/L)	6.01 (1.91, 18.39)	6.87 (1.36, 17.98)	0.656
Hemoglobin (g/L)	149 (99.0, 190.6)	133 (91.5, 182.0)	0.066
Platelet (×109/L)	265.81±128.97	181.38±116.54	0.001
ALT (U/L)	10 (3.0, 44.0)	14 (3.0, 580.8)	0.001
AST (U/L)	37 (14.1, 191.4)	48 (15.8, 1791.2)	0.003
LDH (U/L)	405 (212, 1269)	515 (235, 3512)	0.050
DB (μmol/L)	8.4 (4.9, 23.5)	10.8 (4.4, 179.7)	0.072
UREA (mmol/L)	3.55 (1.38, 9.82)	5.25 (2.91, 18.42)	<0.001
CREA (μmol/L)	44 (18.8, 98.0)	55.5 (14.7, 145.3)	0.050
PT (s)	13.7 (10.8, 23.0)	17.4 (11.6, 31.3)	<0.001
INR	1.18 (0.93, 2.01)	1.51 (1.00, 2.89)	<0.001
APTT (s)	52.5 (33.9, 110.6)	57.6 (32.9, 112.3)	0.259
TT (s)	18.5 (15.2, 27.8)	19.4 (15.2, 54.2)	0.001
ALB (g/L)	33.4 (25.5, 40.1)	31.2 (21.4 to 36.6)	0.001

ALB: albumin; ALT: alanine aminotransferase; APTT: activated partial thromboplastin time; AST: aspartate aminotransferase; CRP: C-reactive protein; CREA: creatinine; INR: international normalized ratio; PT: prothrombin time; TT: thrombin time; UREA: urea nitrogen.

### Independent predictors of 30-day mortality

To further select independent factors of 30-day mortality, we conducted univariate and multivariate analyses on these ­variables with p<0.1 (Table 1). After adjusted these factors (culture ­positive, hemoglobin, platelet, ALT, AST, LDH, DB, UREA, CREA, PT, INR, TT, ALB), hemoglobin>133 g/L (hazard ratio (HR): 0.351, 95% confidence interval (CI): 0.128-0.961, p=0.042) and PT >16.6 s (HR: 4.140, 95%CI: 1.523-11.260, p=0.005) were independent risk markers of 30-day mortality (Table 2). Based on these results, a predictive model was built as follows:

**Table 2:** Analysis of in-hospital death.

Variables	Univariate analyses			Multivariate analyses			
	HR	95%CI	p-value	β	HR	95%CI	p-value
Culture positive	2.695	0.891-8.130	0.076				
Hemoglobin>133 g/L	0.350	0.157-0.782	0.011	-1.046	0.351	0.128-0.961	0.042
Platelet>244 ×109/L	0.210	0.085-0.519	<0.001				
ALT>32 U/L	5.775	2.163-15.415	0.001				
AST>41 U/L	2.104	0.850-4.769	0.133				
UREA>2.9 mmol/L	13.378	1.771-101.045	0.001				
PT>16.6s	4.613	2.029-10.490	<0.001	1.421	4.140	1.523-11.260	0.005
INR>1.45	4.183	1.846-9.474	0.001				
TT >18.35s	2.786	1.129-6.876	0.026				
ALB>33.9 g/L	0.204	0.068-0.612	0.002				
CREA>71 mmol/L	2.991	1.242-7.205	0.018				
LDH>387 U/L	0.905	0.860-0.952	0.134				
DB>13.4 μmol/L	1.955	0.433-8.828	0.538				
Logit p=1.421×PT-1.046×hemoglobin-2.155							

ALB: albumin; ALT: alanine aminotransferase; APTT: activated partial thromboplastin time; AST: aspartate aminotransferase; CI: confidence interval; CREA: creatinine; HR: hazard ratio; INR: international normalized ratio; PT: prothrombin time; TT: thrombin time; statistically significant p-values are denoted in bold (P<0.05).

Logit p=1.421×PT-1.046×hemoglobin-2.155.

### Performance of the predictive model in the prediction of 30-day mortality

**Figure 1:**
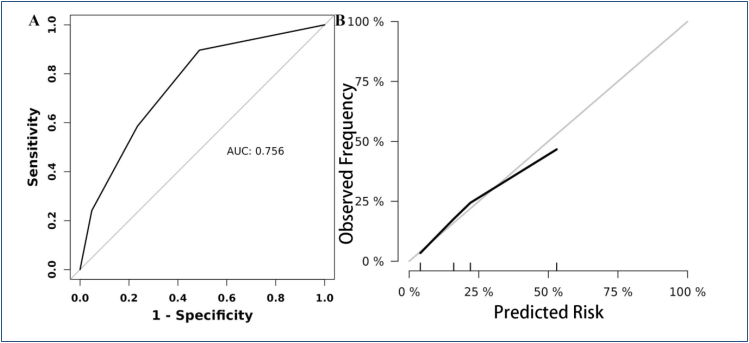
Performance of the predictive model. (A) Receiver operating characteristic curves of factors for predicting mortality. (B) Calibration curves.

Sensitivity and specificity were determined to compare the ­performance of the predictive model and independent ­predictors. Figure 1A shows the ROC curve of the predictive model; the predictive model had the highest area under the curve (AUC) (0.756, 95%CI 0.666-0.847, P<0.001). The calibration curve of the predictive model had demonstrated good ­agreement (Figure 1B). Details of the performance are shown in Supplementary Table 1.

In addition, the patients were divided into two groups based on the predictive model. A comparison was made between the two groups; the results showed that the 30-day mortality rate was higher in the predictive model value>0.05588 groups than that in the predictive model value ≤ 0.05588 groups (24.3 vs. 3.40%).

## DISCUSSION

In this study, we found a significant association among HB, PT, and the risk of 30-day mortality. Based on independent risk factors, we built a predictive model with good performance. In addition, we found that the 30-day mortality rate was higher in the predictive model value>0.05588 group than that in the predictive model value ≤ 0.05588 group.

Neonatal sepsis refers to a severe bloodstream ­infection that occurs in neonates. It is often accompanied by a characterized ­systemic inflammatory response syndrome^12^. Inflammation plays a vital role in the pathogenesis and progression of ­neonatal ­sepsis^13^. During neonatal sepsis, the presence of pathogens triggers an immune response, leading to the release of ­various ­pro-inflammatory molecules such as cytokines, chemokines, and acute-phase reactants^14,15^. However, the overproduction of ­pro-inflammatory molecules can result in widespread ­tissue ­damage, organ dysfunction, and complications ­associated with sepsis^16^.

Hemoglobin is a major component of red blood cells, and its measurement provides information about anemia and ­oxygenation status^17^. In neonatal sepsis, the infection and inflammatory response can impact the hematopoietic system, potentially leading to the development of anemia^18^. The release of inflammatory mediators and activation of inflammatory cells may suppress red blood cell production or promote their destruction, thereby ­reducing ­hemoglobin levels. Furthermore, sepsis can also cause ­systemic hemodynamic changes such as tissue ­hypoperfusion and ­circulatory disturbances, which can influence blood ­hemoglobin levels^19^. In line with prior studies, our study also confirmed that HB was an independent index. Therefore, ­monitoring ­hemoglobin levels in neonates with sepsis can provide ­valuable information about the severity of anemia, inflammatory response, and overall circulatory status. This aids clinicians in assessing the disease severity, ­guiding treatment strategies, and ­monitoring treatment effectiveness.

Prothrombin time, a test that measures the duration taken for the blood to clot, is an important indicator of coagulation function and can provide insights into the body's ability to form blood clots^20^. In neonatal sepsis, the inflammatory response and activation of coagulation pathways can lead to alterations in the coagulation system^21^. Sepsis-induced changes in the ­levels of coagulation factors, platelets, and endothelial cells can affect the clotting process and prolong PT^22,23^. Additionally, ­disseminated intravascular coagulation, a severe complication associated with sepsis, can further disrupt the coagulation ­cascade and contribute to abnormal PT results^24^. By monitoring PT in neonates with sepsis, clinicians can gain insights into the coagulation status and identify potential clotting abnormalities. This information is crucial for guiding appropriate treatment strategies, such as the administration of blood products or anticoagulants, and improving patient outcomes. Our study also confirmed this.

For clinical application, it is important to make the ­assessment of risk factors as convenient as possible. In our study, HB and PT are prevalent in clinical practice and ­convenient to acquire. They were independent factors ­demonstrated by ­multivariate analysis and we built a simple, convincing, and readily ­available model with good performance. On ­subgroup analysis, the 30-day mortality rate was higher in the ­predictive model value>0.05588 groups than that in the ­predictive model value ≤ 0.05588 groups. Identifying high-risk patients may help ­clinicians improve the treatment ­efficacy and clinical outcome.

This study is limited by its retrospective nature and ­single-center data, which may cause selection bias. Second, only ­admission biomarkers were included in the ­present ­analyses, and it is ­possible that dynamic changes in ­biomarkers during the course of treatment might also influence ­outcomes in ­neonatal sepsis. Third, there were no test results for ­inflammatory ­factors, such as procalcitonin, and ­interleukin-6 (IL-6). Fourth, there are no external and internal cohorts. Thus, before ­clinical ­application, large, multicenter, ­prospective ­studies and ­validation cohort need to be conducted to ­determine the value of the predictive model.

## CONCLUSION

Based on the clinical risk factors identified in this cohort, we established a model that can objectively and ­accurately ­predict individualized risk of 30-day mortality. The ­predictive model should help clinicians to improve individual ­treatment, make clinical decisions, and guide follow-up management strategies.

## Data Availability

The datasets are available from the corresponding author on reasonable request.
