# ERRATUM

**DOI:** 10.1590/1806-9282.20231561ERRATUM

**Published:** 2025-06-16

In the manuscript "A practical predictive model to predict 30-day mortality in neonatal sepsis", https://doi.org/10.1590/1806-9282.20231561, published in the Rev Assoc Med Bras. 2024;70(7):e20231561, on page 1:

**Where it reads:**

Tengfei Qiao^1^*, Xiangwen Tu^2^.

**It should read:**

Tengfei Qiao^1^, Xiangwen Tu^2^*

